# Is targeting autophagy mechanism in cancer a good approach? The possible double-edge sword effect

**DOI:** 10.1186/s13578-021-00570-z

**Published:** 2021-03-20

**Authors:** Su Min Lim, Ezanee Azlina Mohamad Hanif, Siok-Fong Chin

**Affiliations:** UKM Medical Molecular Biology Institute (UMBI), Universiti Kebangsaan Malaysia, Jalan Yaacob Latiff, Bandar Tun Razak, Cheras, W. Persekutuan, 56000 Kuala Lumpur, Malaysia

**Keywords:** LRG1, Autophagy, Cancer, Treatment, Precision Medicine

## Abstract

Autophagy is a conserved cellular process required to maintain homeostasis. The hallmark of autophagy is the formation of a phagophore that engulfs cytosolic materials for degradation and recycling to synthesize essential components. Basal autophagy is constitutively active under normal conditions and it could be further induced by physiological stimuli such as hypoxia, nutrient starvation, endoplasmic reticulum stress,energy depletion, hormonal stimulation and pharmacological treatment. In cancer, autophagy is highly context-specific depending on the cell type, tumour microenvironment, disease stage and external stimuli. Recently, the emerging role of autophagy as a double-edged sword in cancer has gained much attention. On one hand, autophagy suppresses malignant transformation by limiting the production of reactive oxygen species and DNA damage during tumour development. Subsequently, autophagy evolved to support the survival of cancer cells and promotes the tumourigenicity of cancer stem cells at established sites. Hence, autophagy is an attractive target for cancer therapeutics and researchers have been exploiting the use of autophagy modulators as adjuvant therapy. In this review, we present a summary of autophagy mechanism and controlling pathways, with emphasis on the dual-role of autophagy (double-edged sword) in cancer. This is followed by an overview of the autophagy modulation for cancer treatment and is concluded by a discussion on the current perspectives and future outlook of autophagy exploitation for precision medicine.

## Introduction

Cells are naturally safeguarded by an efficient check-and-balance mechanism better known as cellular homeostasis to maintain the balance of a wide array of biochemical factors and processes. Among the vital processes, protein synthesis and break down are both essential in maintaining cellular homeostasis for optimal biological activity. For eukaryotic cells, the two major protein degradation pathways are the ubiquitin–proteasome pathway (UPP) and the lysosomal-autophagy pathway [1, 2]. Autophagy is a highly conserved and tightly regulated process where it involves the catabolism of dysfunctional proteins such as senescent organelles, misfolded proteins and intracellular pathogens [3, 4]. In response to stressful conditions including nutrient starvation and hypoxia, autophagy is enhanced to degrade intracellular components and recycle the macromolecule precursors (amino acids, fatty acids and nucleotides) to preserve cellular turnover and homeostasis [4, 5]. To date, autophagy can be classified into three types: macroautophagy, chaperon-mediated autophagy (CMA) and microautophagy [6]. Among them, macroautophagy is the most extensively studied and the term “autophagy” typically refers to macroautophagy, unless otherwise stated [6, 7].

## Mechanism of autophagy

**Fig. 1:**
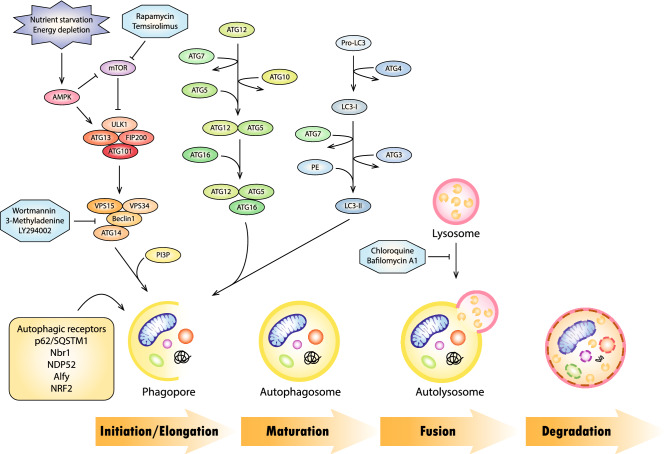
Mechanism of autophagy. The autophagy-related genes involved in the sequential process of autophagy mechanism, from initiation to degradation

Autophagy is a sequential process that involves initiation, elongation, maturation, fusion and degradation [4]. These distinct steps are governed by a series of autophagy-related genes (ATGs) and the dysregulation of ATGs would impact autophagy [4, 8]. Upon induction of autophagy, unc-51-like kinase 1 (ULK1) complex (comprising ULK1, ATG13, focal adhesion kinase family interacting protein of 200 kDa (FIP200) and ATG101) translocates to the phagophore initiation site where it becomes activated through dephosphorylation [9]. Activated ULK1 complex serves as a scaffolding unit to recruit class III phosphatidylinositol 3-kinase (PI3KIII) complex that consist of vacuolar protein sorting 34 (VPS 34), Beclin-1, VPS15 and ATG14-like (ATG14L) [9, 10]. Subsequently, this complex stimulates the synthesis of phosphatidylinositol 3-phosphate (PI3P), a component rich in autophagosomal membranes or phagophores [4, 9]. The autophagy-related genes involved in the sequential process of autophagy mechanism, from initiation to degradation are shown in Fig. 1.

Phagophore, also known as isolation membrane, is a sack-like structure that matures into autophagosome and fuses with the lysosome to form autophagosome [7]. The double membrane of phagophores may originate from endoplasmic reticulum, mitochondria, Golgi complex or other organelles [11]. Phagophores act by engulfing senescent cytosolic components and subsequently elongates into spherical autophagosomes under the modulation of two ubiquitin-like conjugation pathways: ATG12 conjugation system and ATG8 conjugation system [9, 12]. In the ATG12 conjugation system, both E1-like enzyme ATG7 and E2-like enzyme ATG10 facilitate the conjugation of ATG12 to ATG5, forming ATG12-ATG5 complex [13]. The ATG12-ATG5 complex directly associates with ATG16 and binds to the autophagosomal membrane [14]. The dissociation of ATG12-ATG5-ATG16 complex from the membrane following successive formation of autophagosome allows it the be identified as a marker for early steps of autophagy [10]. Meanwhile, the mammalian orthologues of the ATG8 can be categorized into the microtubule-associated protein 1 light chain 3 (LC3), γ-aminobutyric-acid type A receptor-associated proteins (GABARAPs) and Golgi-associated ATPase enhancer of 16 kDa (GATE-16) subfamilies based on their amino acid sequence homology [15]. Both LC3 and GABARAP subfamilies are indispensable for functional autophagy: LC3s function in phagophore elongation whereas the GABARAP/GATE-16 subfamilies engage in the closure and maturation of autophagosomes [16]. The abundance of ATG8 controls the size (or volume) of autophagosomes but does not affect the number of autophagosomes nor the frequency of autophagosome formation [17]. Upon autophagy induction, LC3 translocates from the nucleus to the cytosol to engage with autophagosomes [18, 19]. Subsequently, the precursor proLC3 is cleaved by ATG4 into LC3-I and conjugated with phosphatidylethanolamine (PE) phospholipid by ATG7 and ATG3 along with the ATG12-ATG5-ATG16 complex to form LC3-II [12, 20, 21]. The soluble LC3-I is localized in the cytoplasm whereas the lipidated LC3-II is attached to the inner and outer sides of the autophagosome membranes [21–23]. Due to the abundance of LC3 in autophagosome membranes, it is widely used as a marker for assessing autophagy [24]. Besides autophagosome biogenesis, the ATG8 proteins recognize autophagic receptors (such as p62/SQSTM1, neighbour of Brca1 (Nbr1), nuclear dot protein 52 kDa (NDP52), autophagy-linked FYVE protein (Alfy) and NRF2) through LC3-interacting region (LIR) motif and target them for autophagic degradation [22, 25]. Following elongation and maturation, ATG8 is released from autophagosomes by deconjugation through the action of ATG4 [17]. Then, the sealed autophagosome merges with lysosome and form autolysosome [7]. The formation of autolysosome releases sequestered autophagic bodies and the inner membrane into the lumen where they are exposed to acidic hydrolases and lipases for degradation [7]. The subsequent macromolecules including amino acids, fatty acids and nucleic acids are then, recycled back into the cytosol by permeases such as ATG22 [26]. This process allows the biosynthesis of essential components required during critical conditions, such as stress and greatly improves cell survival in a check-and-balance manner [27].

## Pathways controlling autophagy

Under normal physiological conditions, autophagy occurs at a basal rate to maintain cellular viability and homeostasis [28, 29]. Upon disruption by environmental stress (such as nutrient starvation, endoplasmic reticulum (ER) stress, hypoxia and drugs), autophagy is modulated for adaptation and survival by several pathways including mammalian target of rapamycin (mTOR), AMP-activated protein kinase (AMPK), Wnt and TGFβ [29–31].

The mammalian orthologue of yeast TOR protein, mTOR, plays a crucial role in regulating autophagy by sensing intracellular stress and environmental factors [29, 30]. As a negative regulator of autophagy, mTOR integrates signals from several upstream molecules including AMPK and PI3K [30, 32, 33]. The mTOR constitutes of two distinct complexes, the mTOR complex 1 (mTORC1) and mTOR complex 2 (mTORC2) [34, 35]. Function-wise, mTORC1 responds to nutrient levels whereas mTORC2 is influenced by growth factors [34]. Activation of mTORC2 can also be achieved by the presence of amino acids via the PI3K/Akt signalling [36]. When nutrient is sufficient, mTORC1 is activated and autophagy will be inhibited [37]. In contrast, mTORC1 is inactivated during nutrient depletion, causing induction of autophagy to mobilize the available macromolecules [37–40]. During this response, the inhibition of mTORC1 will activate the ULK1 complex to drive the downstream activation of autophagy [41]. In addition to the nutrient-sensing role, mTOR also partially regulates autophagy in response to growth factors and hypoxia [6, 37, 42].

AMP-activated protein kinase (AMPK) acts as an energy-sensing kinase that promotes autophagy by detecting the abundance of AMP and ATP [43, 44]. In response to energy starvation, AMPK is activated by calcium/calmodulin-dependent protein kinase kinase β (CaMKKβ) and liver kinase B1 (LKB1), through phosphorylation at Thr-172 residue [45–47]. Furthermore, ADP allosterically activates AMPK whereas AMP protects AMPK from dephosphorylation, which is crucial for AMPK activation [44]. Activated AMPK phosphorylates the tuberous sclerosis complex (TSC) and attenuates mTOR activity leading to induction of autophagy [6, 48, 49]. Moreover, elevated intracellular calcium concentration induced by ER stress may also promote autophagy through activation of AMPK [6, 50].

Besides that, a regulatory feedback between autophagy and Wnt/β-catenin signalling has been reported [51]. β-catenin negatively modulates autophagy by reducing autophagosome formation and LC3-II puncta during starvation and nutrient-rich conditions [52]. Interestingly, p62 protein expression is increased with β-catenin knockdown while autophagic flux is not hindered [52]. Further investigation revealed that β-catenin represses transcriptional expression of p62 through binding of transcription factor TCF4 [52]. In the crosstalk of autophagy and Wnt/β-catenin signaling, β-catenin integrates growth and stress signals to coordinate proliferation and autophagy. As only a basal level of autophagy is required under normal conditions, β-catenin limits autophagy and represses p62 transcription [52]. However, when nutrient is depleted, β-catenin permits autophagy activation, relieves suppression on p62 transcription and β-catenin is degraded by autophagy [52].

Meanwhile, the transforming growth factor beta (TGFβ) signalling pathway regulates a plethora of biological functions including cell proliferation, differentiation, migration and adhesion to maintain cellular homeostasis [53]. In the context of autophagy, TGFβ signals through both SMAD and non-SMAD pathways (TAK1/MKK3 and JNK pathways) to promote the formation of autophagosome and conversion of LC3-I to LC3-II [54–56]. In renal carcinoma cells, the supplementation of TGFβ has been reported to augment the expression of autophagy markers, LC3-II and Beclin-1 [57]. The enhanced autophagy activation by TGFβ results in increased secretion of lactate that mediates TGFβ autocrine [54]. Of note, autophagy activation may in turn enhances TGFβ expression, thus forging a positive feedback loop in cancer progression [58, 59].

The cAMP-dependent protein kinase A (PKA) is also capable of controlling autophagy. PKA responds to glucose and carbon levels. When glucose level is high, PKA inhibits autophagy directly by phosphorylating mTORC1 or indirectly through inhibition of AMPK [60]. Nonetheless, other stimuli such as lipid accumulation and iron depletion may also regulate autophagy [61, 62]. The interconnection and signalling crosstalk between various stimuli are indeed very sophisticated and is of major interest to further elucidate the captivating autophagy mechanism.

## Autophagy in cancer

Autophagy is an evolutionarily conserved process for maintaining cellular homeostasis. By recycling macromolecule precursors to supply nutrient source and building blocks, autophagy is associated with cell survival [5]. However, uncontrolled persistent activation of autophagy may lead to cellular disintegration and ultimately cell demise [63, 64]. The dysregulation of autophagy has been implicated in several diseases including neurodegenerative diseases [64, 65], infectious diseases, malignancies of liver, colorectal, gastric, breast, ovarian and many more [66]. In cancer cells, the role of autophagy is rather controversial as it prevents malignant transformation and conversely promotes tumour growth. This discrepancy ignites debates over the exact role of autophagy, either a friend or a foe in the perspective of cancer.

**Fig. 2:**
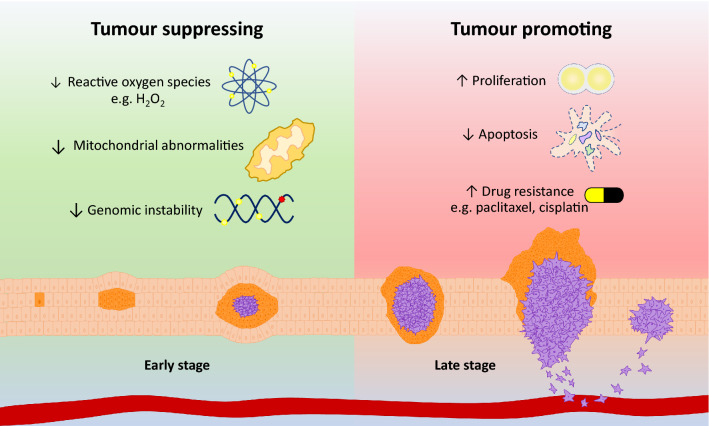
The role of autophagy in cancer progression. Basal autophagy plays a protective role in maintaining homeostasis under normal conditions and early stage of cancer. During cancer development, autophagy aids to overcome stressful stimuli such as hypoxia and nutrient deprivation. Subsequently, autophagy supports cancer cell growth and facilitates malignant progression in established tumours

Autophagy was previously thought to play a protective role against cancer development, as evidenced by the monoallelic deletion of Beclin-1 and autophagy inactivation in breast, ovarian and prostate cancer [67–70]. Ovarian carcinoma patients with a high expression of Beclin-1 were found to have a better prognosis, suggesting that autophagy might limit cancer progression [71]. Furthermore, a reduced expression of autophagy genes (ATG5, ATG7 and Beclin-1) has been observed in hepatocellular carcinoma (HCC) cells. Of note, Beclin-1 expression was significantly decreased in the HCC tissues compared to the adjacent non-tumour tissues [72]. Conversely, the basal level of autophagy was enhanced in melanoma patients with increased autophagosome puncta and LC3-II levels [73]. In colorectal cancer (CRC), the expression of LC3 in tumour tissues is significantly higher than the control, indicating an elevated autophagy activity. More importantly, the expression of LC3 correlated with tumour aggressiveness and thus suggesting a tumour-promoting role of autophagy [74]. Despite the reduced expression of Beclin-1 in a subgroup of CRC, the overexpression of Beclin-1 in CRC was significantly correlated with nodal involvement, high histological grade and vascular invasion [75]. Similarly, Beclin-1 expression in gastric cancer has gained conflicting results. On one hand, it was found that Beclin-1 expression was increased in gastric carcinomas whereas the other observed a decreased expression compared to adjacent non-tumour tissue [76, 77]. Taken together, these pieces of evidence suggest the equivalently important roles of autophagy in both tumour suppressing and promoting activities, hence confers the double-edged sword tag (Fig. 2).

## Autophagy as tumour suppressor

During normal conditions and early stage of cancer, autophagy serves as a shield to protect cells from harmful stimuli and malignant transformation. By limiting the devastating effect of reactive oxygen species (ROS), autophagy prevents DNA damage and maintains genome integrity [66, 78]. Upon starvation, the production of ROS triggers autophagy, specifically, H_2_O_2_ reversibly modifies the cysteine residues of ATG4 and thereby disrupts the active site required for delipidation of LC3 [79, 80]. This results in accumulation of lipidated LC3 and increased autophagosome formation [79]. Furthermore, the scavenger role of autophagy is evidenced by the accumulation of ROS in autophagy-deficient cervical cancer cells [79]. On the other hand, inhibition on autophagy renders the immortalized mouse kidney (iBMK) cells to be susceptible to mutations and chromosomal instability that may result in aneuploidy [78]. Moreover, the autophagy-defective iBMK cells that suffer metabolic stress exhibit a build-up of p62 along with damaged mitochondria and ER chaperones, indicating the failure of protein removal [81]. The accumulation of p62 in turn promotes ROS production and triggers DNA damage response which ultimately contributes to tumourigenesis [81]. In contrast, autophagy suppresses tumourigenesis by eliminating toxic mutagens and avoiding the accumulation of genetic defects [82]. This mechanism also prevents excessive inflammation and induces senescence to hinder the growth of tumour cells [66]. As a negative regulator of the nod-like receptor family pyrin domain containing 3 (NLRP3) inflammasome, autophagy reduces inflammation in fetal human colon cells [83]. Furthermore, reduced autophagy in renal cell carcinoma promotes cell proliferation suggesting that autophagy is required to constrain the growth of cancer cells [84].

## Autophagy as tumour promoter

As the tumour develops and progresses, autophagy, in turn, fuels and supports the growth of cancer cells. Due to the increased metabolic demand of highly proliferative cancer cells and poor vascularization in solid tumours, the tumour microenvironment is often hypoxic and nutrient-deprived, that may trigger autophagy for an adaptive metabolic response [85–87]. Through recycling macromolecules and supplying building blocks, autophagy contributes to the survival of tumour cells under these unfavourable stress conditions [85, 88].

Autophagy facilitates cancer progression by promoting the migration and invasion capacity of cancer cells. Upon starvation, autophagy promotes the invasion and epithelial-mesenchymal-transition (EMT) of the hepatocellular carcinoma cells [59]. The induced autophagy in hepatocellular carcinoma was also reported to support pulmonary metastasis by promoting anoikis resistance and colonization [89]. Besides that, hypoxia-induced autophagy may also protect hepatocellular carcinoma cells from apoptosis during nutrient deprivation via Beclin-1 dependent pathway [90]. In pancreatic cancer, hypoxia-induced autophagy enhanced migration and invasion through HIF-1α upregulation and EMT [91]. The inhibition of autophagy by shRNA targeting ATG12 in a glioma 3D organotypic model has been shown to impair cell invasion but does not affect cell viability, proliferation and cell migration [92]. Moreover, autophagy induction promoted migration and invasion of bladder cancer cells by facilitating EMT via TGFβ pathway [58]. Similarly, the invasion capacity of hepatocellular carcinoma induced by autophagy is dependent on TGFβ signalling and EMT [59]. Accordingly, silencing of autophagy-related genes or treatment with autophagy inhibitors abrogated EMT and reduced the invasiveness of hepatocellular carcinoma cells during starvation [59]. Furthermore, genetic inhibition of autophagy in RAS-activated cells inhibits the formation of invasive protrusions, maintains the integrity of basement membrane and restricts ECM proteolysis [93]. Treatment of conditioned media from autophagy competent RAS-activated cells rescued the migration and invasion capability of autophagy-deficient RAS-activated cells, suggesting that autophagy is required for the secretion of pro-migratory cytokines, namely IL6 [93]. Intriguingly, autophagy is inversely correlated with migration and invasion in glioblastoma cell line and also in primary cells [94]. It is also notable that autophagy is capable of inducing or inhibiting EMT and interestingly EMT could also activate or represses autophagy [3].

In addition, autophagy is essential in cancer stem cells for dictating their pluripotency, self-renewal and drug resistance [95–98]. The expression of Beclin-1 and subsequent autophagy activation is necessary for the maintenance and tumourigenicity of breast cancer stem cells [99]. The autophagy associated factors, DRAM1 and p62, have also been found to regulate the energy metabolism and invasion of glioma stem cells through activation of autophagy, whereas the knockdown of the autophagy-related gene, ATG12, was reported to compromise the invasive capability of the tumour cells in an organotypic model of glioma cells [92, 100]. The enhanced autophagy flux in ovarian cancer stem cells supports self-renewal and chemoresistance through upregulation of the transcription factor Forkhead Box A2 (FOXA2) [95]. It was evidenced that inhibition of autophagy in ovarian cancer stem cells decreased the size and number of sphere formation, reduce the population of CD24^−^ and CD44^+^ cells, increased drug sensitivity to paclitaxel and attenuates the expression of FOXA2 [95]. Chemotherapy has been reported to promote the proportion of CD133^+^ cancer stem cells, which show higher autophagy level, in non-small cell lung carcinoma [101]. Upon inhibition of autophagy, lung cancer stem cells manifest reduced sphere formation and colony formation [101]. Combined treatment of autophagy inhibitor and chemotherapy greatly improved the efficacy of chemotherapy by reducing the population of CD133^+^ cancer stem cells in vitro and impede tumour growth in vivo [101]. Similarly, the inhibition of autophagy has been found to improve the sensitivity of colorectal cancer stem cells to photodynamic therapy [92]. In gastric cancer stem cells, enhanced autophagy contributed to chemoresistance through Notch signalling pathway [96]. Another role of autophagy in maintaining cancer stem cells is by regulating CD24 expression and IL6 secretion [97]. In breast cancer model of MCF7 and MDA-MB-468, autophagy-deficient cells restore mammosphere formation with the supplementation of IL6 or treatment of conditioned media from autophagy competent cells, suggesting that autophagy is required for the secretion of IL6 to maintain cancer stem cells [97]. Furthermore, basal autophagy is crucial in maintaining pluripotency of cancer stem cells and any deviation from basal level of autophagy, either activation or inhibition, may promote differentiation and senescence [98]. As depicted in teratocarcinoma stem cells, both induction and suppression of autophagy reduce cell viability, proliferation and pluripotency while differentiation is promoted [98].

In short, the function of autophagy in cancer is context-dependent and highly influenced by the tumour microenvironment, disease stage and exposure to external stimuli. The controversial role of autophagy in cancer warrants further investigation to unravel its therapeutic potential as a cancer drug target.

## Modulation of autophagy in cancer

Over the last decade, autophagy has emerged as a promising target for cancer therapy. However, the opposing roles of autophagy in promoting and suppressing tumour growth have presented a major challenge in modulating autophagy for cancer therapy. Despite that, several autophagy modulators have been approved by the U.S. Food and Drug Administration (FDA) for cancer treatment and numerous are currently in clinical trials [102]. Interestingly, some reports suggest a synergistic effect on the use of autophagy inhibitors and other therapeutic agents. We summarised the available data collected from previous in-vitro and pre-clinical studies on various malignancies in Table 1.

**Table 1:** Genetic and pharmacological inhibition of autophagy synergize with therapeutic agents in various malignancies

Types of cancer	Model	Therapeutic agent	Autophagy inhibitor		Outcomes/Effects/Phenotypes	
			Pharmacologic	Genetic		
Chemotherapy						
B cell lymphomas	Mice	Cyclophosphamide	Chloroquine Hydroxychloroquine	ATG5 shRNA	Complete tumor regression and delayed tumor recurrence	[103]
Brain cancer	AM38 and 794 cells	Vemurafenib, Vinblastine	Chloroquine	–	Improved tumor cell kill	[104]
Esophageal squamous cell carcinoma (ESCC)	EC9706 cells	5-FU	LY294002 (LY)	–	Improved the sensitivity of cancer cells towards 5-FU	[105]
Esophageal squamous cell carcinoma (ESCC)	EC9706 cells	Cisplatin	3-methyladenine	–	Enhanced cisplatin-induced cell death and cell cyle arrest	[106]
Colorectal cancer	HT29	5FU	Chloroquine	–	Reduced proliferation and cell growth, potentiated cell cycle arrest	[107]
Colorectal cancer	SW480 and SW620	Oxaliplatin	–	ATG5, ATG7, shRNA	Decreased cell viability and promoted chemotherapy efficacy	[108]
Glioma	U373-MG cells	Temozolomide	Bafilomycin A1	–	Suppressed proliferation and induced apoptosis	[109]
Lung cancer	A549 cells	Paclitaxel Cisplatin	3-methyladenine	–	Enhanced cytotoxic effect of chemotherapy and promoted apoptosis	[110]
Lung cancer	A549 cells	Cisplatin	3-methyladenine	–	Inhibited proliferation, induced apoptosis and increased chemosensitivity	[111]
Lung cancer	A549 cells, mice	Cisplatin	Chloroquine	–	Improved efficeincy o f chemotherapy and suppressed tumour growth, reduced percentage of cancer stem cells	[101]
Myeloid leukemias	K562 cells	Daunorubicin	Chloroquine U0126	ATG5, ATG7, Beclin-1 siRNA	Promoted chemotherapy efficacy	[112]
Ovarian cancer	3AO and SKOV3	Paclitaxel	Chloroquine	ATG5 shRNA	Decreased self-renewal ability of cancer stem cells	[95]
Pancreatic cancer	Mice	Gemcitabine	Chloroquine	ATG5, ATG7, Beclin-1 shRNA	Suppress cancer stem cells activity, cancer cell growth and tumour formation	[113]
Pancreatic cancer	PANC-1, BxPC-3	5FU, Gemcitabine	Chloroquine	–	Potentiated growth-inhibitory effects	[114]
Renal cancer	ACHN-5968, UOK257 cells	Paclitaxel	3-methyladenine	Beclin 1 siRNA	Enhanced paclitaxel-mediated cytotoxicity and apoptosis	[115]
Other therapies						
B cell lymphomas	Mice	ER signalling inhibitor, Tamoxifen	Chloroquine Hydroxychloroquine	ATG5 shRNA	Complete tumor regression and delayed tumor recurrence	[103]
Bladder cancer	J82 and T24 cells	AR signaling inhibitor, Enzalutamide	3-methyladenine Bafilomycin A1 Chloroquine	ATG5 shRNA	Triggered apoptosis and inhibited proliferation	[116]
Bladder cancer	UMUC3 cells, mice	AR signaling inhibitor, Enzalutamide	Chloroquine	–	Impaired tumour growth and improved therapeutic sensitivity	[116]
Bladder cancer	EJ and T24 cells, mouse	Radiation	Chloroquine	–	Promotes radiosensitivity and induced apoptosis	[117]
Cervical carcinoma	HeLa cells	Photodynamic therapy, Photofrin	–	sgATG5	Enhanced apoptosis and protein carbonylation	[118]
Colorectal cancer	SW480 cells	PI3K-mTOR inhibitor, NVP-BEZ235	3-methyladenine Chloroquine	–	Reduced cell viability and enhanced apoptosis	[119]
Lung Cancer	A549, NCI-H1299, SKMES-1 cells	EGFR inhibitor, Gefitinib, erlotinib	Chloroquine	ATG5, ATG7 siRNA	Augmented growth inhibition	[120]
Melanoma	A2058, C8161, SKMEL2, UACC903,	mTOR inhibitor, Temsirolimus	Hydroxychloroquine	–	Impaired cancer cell growth and increased cell death	[73]
Oral squamous cell carcinomas	KB cells, mice	Cytokine, IL24	3-methyladenine	–	Promoted apoptosis, attenuated tumour growth	[121]
Renal cell carcinoma	RCC4 cells, mice	mTOR inhibitor, Temsirolimus	Chloroquine	ATG7 shRNA	Improved antitumour activity	[122]
Renal cell carcinoma	A498	mTOR inhibitor, Temsirolimus	Chloroquine	–	Enhanced cycotoxicity and apoptosis	[57]

*ER* estrogen receptor, *AR *androgen receptor

Metformin, the most commonly prescribed anti-diabetic drug was found to impair tumour growth in melanoma and cervical cancer by promoting autophagy via AMPK activation [123, 124]. AMPK serves as a sensor of cellular energy and promotes autophagy when the AMP/ATP ratio is increased [125]. The mechanism of action by AMPK towards autophagy is either directly by phosphorylation of ULK1 or indirectly through inhibition of mTOR complex activities [126, 127].

The negative regulator of autophagy, mTOR, has been extensively studied as a therapeutic target for autophagy modulation. As mTOR inhibits autophagy, mTOR inhibitors have been developed to induce autophagy. Rapamycin (also known as sirolimus) is an mTOR inhibitor that promotes autophagy through binding with FK506-binding protein 12 (FKBP12) and stabilizing the raptor-mTOR complex, thereby repressing the action of mTOR [128]. The treatment of neuroblastoma cells with rapamycin has been found to inhibit proliferation through autophagy induction and cell cycle arrest [129]. Furthermore, a recent study in murine sarcoma cells suggested that the tumour suppressive effect of rapamycin results from successive autophagy and depletion of the cancer stem cells [130]. Of note, mTOR is central to diverse biological pathways including immune regulation, cell cycle progression, protein synthesis and angiogenesis. Thus, targeting mTOR with rapamycin and its derivatives (rapalogs) may affect other metabolic processes as well [131].

Besides the canonical mTOR dependent pathways, various drugs induce autophagy in an mTOR-independent manner. These include inositol monophosphatase (IMPase) inhibitors, trehalose, class I PI3K inhibitors and calcium channel blockers that are capable of enhancing autophagy [125, 132, 133]. Physiologically, autophagy is induced in response to metabolic stress and thus starvation along with ER stress inducers could also promote autophagy.

Corresponding to the tumour promoting effect of autophagy, autophagy inhibitors have been characterized to attenuate the tumour growth. As such, autophagy inhibition potentiates the cytotoxicity effect of icaritin in colorectal cancer cells [134]. The upstream molecule of mTOR, PI3K is another attractive molecule for modulating autophagy. Several PI3K inhibitors have been used as autophagy inhibitors including 3-methyladenine (3-MA), wortmannin and LY294002 [135]. The 3-MA exerts its inhibitory effect on breast cancer cells and thereby reducing cell viability [136]. Interestingly, 3-MA has been found to drive autophagy in nutrient-rich conditions, in addition to its suppressive effect during nutrient deprivation [135]. Hence, the use of 3-MA as an autophagy inhibitor must be considered thoroughly. Wortmannin is another PI3K inhibitor that prevents autophagy via persistent blocking of class I PI3K and transiently suppresses the PI3K class III [135]. Owing to the consistent inhibitory action of Wortmannin independent of the nutritional status, it is a more preferable drug for autophagy inhibition [135].

Combined treatment of autophagy modulators with different therapeutic agents has been found to synergistically suppress tumour growth and improve patient response to cancer treatment. In refractory metastatic colorectal cancer, the treatment of antiangiogenic tivozanib along with mTOR inhibitor, everolimus was well tolerated and 50% of the patients continue to have stable disease [137]. Furthermore, the autophagy inhibitor, chloroquine, enhanced chemosensitivity of brain tumours with BRAF V600E mutation and improved the clinical outcome of a patient with drug resistance [104]. However, the synergistic effect was not observed in the BRAF wild-type tumours suggesting that autophagy dependence of tumours is crucial for the administration of autophagy inhibitors [104].

Interestingly, the combination of autophagy inducer, temsirolimus and autophagy inhibitor, chloroquine promotes drug sensitivity and triggers cell death of renal cell carcinoma cells, which are otherwise refractory to treatment [57]. Similarly, concurrent activation and inhibition of autophagy by rapamycin and chloroquine, respectively, act in concert to promote chemosensitization of hepatoma cells through suppression of mTOR and Akt pathway [138]. These data suggest that a drug combination that includes autophagy modulators may be a promising regimen. Besides pharmacological modulators, genetic manipulation of autophagy has also been reported to show a similar result in anti-tumour activity by suppressing proliferation, promoting apoptosis, improved drug sensitivity and inhibiting cancer stem cell activity (Table 1).

The therapeutic potential of autophagy modulation has been contentious and context dependent. Hence, assessing and monitoring autophagy levels in vivo would be crucial in stratifying patients who are likely to respond to autophagy modulation. Theoretically, tumours with higher autophagy activity or autophagy dependency would possibly be more susceptible to autophagy inhibition. Most importantly, some autophagy-related proteins have autophagy-independent roles and thus, autophagy modulation may affect the other biological functions. Of note, autophagy inhibition has a differential effect on cells with varying degree of autophagy dependency, while suppression of autophagy promotes secretion of IL6 in autophagy dependent MCF7 cells, it decreases the expression of IL6 in autophagy dependent MDA-MB-468 cells [97]. Therefore, specific and effective autophagy modulators are needed for improved cancer treatment.

## Current perspectives and future outlook

Autophagy has been reported to have controversial roles in cancer progression. During the early stage of cancer, autophagy plays a protective role to suppress malignant transformation. However, in the established tumour, autophagy supports and enhances tumour growth. This dual function of autophagy in cancer has gained much attention and it is indeed an attractive target for cancer treatment. For tumours with autophagy dependency, autophagy inhibitor would be beneficial. In contrast, tumours with autophagy deficiency would respond to autophagy inducers. It is important to note that there are some parameters to be considered for the application of autophagy modulators in cancer treatment. For instance, the circulating concentration of pharmacological autophagy modulators, the effect and drug toxicity of autophagy modulators in normal tissues, the influence on immune antitumour response and the plausibility of autophagy switch from cytoprotective to nonprotective function [139, 140]. Several factors have been suggested to play a role in autophagy switch including the presence of functional p53, vitamin D treatment, drug sensitivity and different stages of cancer [141]. Depending on the p53 status, radiation-induced autophagy could have distinct functions [141].

In p53 wild type breast cancer cells, radiation-induced autophagy is cytoprotective whereby autophagy inhibition could effectively promote radiation sensitivity [141]. Conversely, radiation-induced non-protective autophagy in breast cancer cells with defective p53 [141]. The dependency of p53 in autophagy switch is not only exclusively in breast cancer, but also appears to be critical in non-small cell lung cancer, pancreatic, colorectal, head and neck cancer [141, 142]. Moreover, it has been reported that the treatment of vitamin D sensitizes breast cancer and non-small cell lung cancer cells to radiation by employing cytotoxic autophagy [143–145]. Interestingly, studies on osteosarcoma and leukemic cells unveil the cytoprotective role of autophagy is in drug-resistant cells whereas drug-sensitive cells displayed cytotoxic autophagy [146, 147]. Thus, autophagy modulators should be used with caution and drugs specifically targeting the autophagy pathway are urgently needed. Although the autophagy mechanism has been largely identified, several other molecules that play a role in autophagy remain to be discovered. Further understanding of the underlying mechanism of autophagy is paramount to elucidate its precise role in cancer.

Another intriguing aspect of targeting autophagy in cancer is the possible intricate crosstalk between the autophagy and apoptosis mechanism. Autophagy and apoptosis are two distinct catabolic pathways that may coordinate or counteract under certain conditions [87, 148]. Autophagy is triggered as an initial response to stress whereas intense and prolonged stress stimuli would induce apoptosis, therefore autophagy often precedes apoptosis [148]. In most of the scenarios, autophagy and apoptosis are inversely regulated, i.e. autophagy induction would prevent apoptosis and conversely apoptosis activation would suppress autophagy [148]. However, it has also been shown that, in some specific circumstances, autophagy or products from autophagic machinery may activate apoptosis to limit tissue damage [148]. Several regulators have been found to control both autophagy and apoptosis, simultaneously, suggesting the potential of limiting the tumour growth by targeting both programmed cell death mechanisms with one stone [148, 149]. In addition to apoptosis, the combination of autophagy modulators with drugs regulating other biological processes is another promising area in treating cancer. To date, numerous studies have been carried out with different combinations of autophagy modulators and chemotherapeutic drugs. These inevitable pieces of evidence provide insight into autophagy modulation as a potential adjuvant in cancer therapeutics.

Autophagy is commonly assessed by observation of autophagy structures and measurement of proteins degraded by the lysosomal activity [150]. As autophagy is a multistep process, static analysis is rather inaccurate and it is unlikely to differentiate between autophagy induction or lysosomal inhibition [150]. Although measuring autophagy flux with specific proteins undergoing autophagic degradation (e.g. LC3 and p62) could provide a precise evaluation of the autophagic activity, it has been reported that some residual autophagy is independent of LC3 and p62 [151, 152]. Hence, a more reliable approach in monitoring autophagy flux is needed to allow efficient and robust monitoring of autophagy activity.

Taken together, autophagy inhibitors would benefit patients with autophagy up-regulation machinery whereas autophagy inducers would be effective for patients with autophagy down-regulation machinery. However, the underlying mechanism of autophagy and the intricate crosstalk between autophagy and apoptosis has not been fully elucidated. Thus, the therapeutic application of autophagy modulators warrants further investigations and specific evaluation.

## Conclusion

Targeting autophagy in precision medicine for cancer is no doubt a very attractive strategy. The exploitation of the knowledge on how some cancer entities suppress the autophagy mechanism that supports their survival and dodge death may indeed turn the tables on cancer. Hence, it is crucial to note that timing is the key for such a purpose, given the controversial role of autophagy in cancer progression. Treatment targeting this mechanism must be given precisely at the right place and time to be beneficial, or else unfortunate catastrophe may be cast. Despite the encouraging results of autophagy modulators, the fastidious condition of autophagy modulation also signifies that autophagy is in fact not a critical target and may not be the most judicious approach, at least as a standalone therapy, to change the tumour evolution due to its paradoxical role, unless the detailed mechanism has been revealed. The molecular mechanism of autophagy is pending for more discovery.

## Data Availability

Data will be provided upon request.
